# Recommendations for the intra-hospital transport of critically ill patients

**DOI:** 10.1186/cc9018

**Published:** 2010-05-14

**Authors:** Benoît Fanara, Cyril Manzon, Olivier Barbot, Thibaut Desmettre, Gilles Capellier

**Affiliations:** 1 Department of Emergency Medicine, Jean Minjoz University Hospital, 25030 Besançon, France

## Abstract

**Introduction:**

This study was conducted to provide Intensive Care Units and Emergency Departments with a set of practical procedures (check-lists) for managing critically-ill adult patients in order to avoid complications during intra-hospital transport (IHT).

**Methods:**

Digital research was carried out via the MEDLINE, EMBASE, CINAHL and HEALTHSTAR databases using the following key words: *transferring*, *transport, intrahospital *or *intra-hospital*, and *critically ill patient*. The reference bibliographies of each of the selected articles between 1998 and 2009 were also studied.

**Results:**

This review focuses on the analysis and overcoming of IHT-related risks, the associated adverse events, and their nature and incidence. The suggested preventive measures are also reviewed. A check-list for quick execution of IHT is then put forward and justified.

**Conclusions:**

Despite improvements in IHT practices, significant risks are still involved. Basic training, good clinical sense and a risk-benefit analysis are currently the only deciding factors. A critically ill patient, prepared and accompanied by an inexperienced team, is a risky combination. The development of adapted equipment and the widespread use of check-lists and proper training programmes would increase the safety of IHT and reduce the risks in the long-term. Further investigation is required in order to evaluate the protective role of such preventive measures.

## Introduction

For over 200 years, from the first Napoleonic wars to the latest international conflicts in Iraq and Afghanistan, military medicine on the battlefield has acted as a catalyst for the development of civilian healthcare. Evacuation and care techniques established when treating the wounded have led to significant advancements in technology and in the human and material resources used in the management and transfer of critically ill patients [1]. Since 1970 [2], the number of international publications in the literature on the analysis and overcoming of risks during the intra-hospital transport (IHT) of critically ill patients has been on the constant increase, particularly over the last fifteen years [3-22].

Several methods of analysis have contributed to the knowledge of IHT-related risks. Epidemiological studies [7,9,10,12,14-16,18] and feedback from intensive care societies [4-6,11,21,23] have contributed to the gathering of a list of Adverse Events (AE) associated with IHT, and to the identification of risk factors (RF) relating to the patient, transport organisation, and technical, human and collective factors.

IHT-related risks can be overcome by developing a common, widespread culture through the standardisation of procedures [4-6,11,21,23], resulting in standard systems of working and a homogenisation of the modalities implemented for IHT.

This step has contributed to a lower AE incidence [14] and to a permanent guarantee that, through diagnostic or therapeutic procedures, the benefits of IHT for the patient outweigh the risks.

However, despite the improvements in IHT practices, AE incidence remains high and constitutes a significant risk for the transport of critically ill patients [14,16]. This review provides an up-to-date presentation of the knowledge acquired over the past 10 years concerning RFs, the incidence and nature of AEs, and the current recommendations for carrying out IHT.

The objective is to provide Intensive Care Units (ICU) and Emergency Departments (ED) with a set of practical procedures (check-lists) for managing critically-ill adult patients in order to avoid complications during IHT.

## Materials and methods

Digital research was carried out via the MEDLINE, EMBASE, CINAHL and HEALTHSTAR databases using the following key words:*transferring*, *transport, intrahospital *or *intra-hospital*, and *critically ill patient*. All English and French publications on the IHT of critically-ill adult patients were analysed and the reference bibliographies of each of the selected articles between 1998 and 15 February 2009, were then studied in order to make our research complete.

## Results

In total, 66 publications were identified, 40 of which were wholly or partly dedicated to IHT. Eight of the publications meet the criteria for epidemiological studies of AEs arising during the IHT of critically-ill adult patients; five are recommendations issued by various intensive care or emergency medicine colleges and societies; and three have a particular emphasis on IHT. Two reviews of the literature on IHT have been carried out by C Waydhas in 1999 [22] and VW Stevenson in 2002 [24]. The other publications include editorials, question/response letters to the Editor and trials evaluating the equipment used for IHT.

Among the eight epidemiological studies focusing on identifying AEs during the IHT of adult patients, six are prospective [9,10,12,14-16], and two are retrospective [7,18]. The number of subjects ranges from 35 to 297, covering between 35 and 452 IHTs from the ED [12,16] or ICU (medical or surgical) [7,9,10,14,15,18], to a different ICU, or to another department for diagnostic (tomodensitometry (TDM), MRI, and so on) or therapeutic (surgery, interventional radiology, and so on) procedures.

The type of AE (clinical or material), the global and specific AE incidence, the number of patients on MV and the composition of IHT teams are summarised in Table 1.

**Table 1:** Summary of epidemiological studies on adverse events during IHT from 1999 to 2007

Author (Year)	Type of study	N° patients Site of origin	N° of IHTs	Destination procedures	Global AE incidence	Cardiovascular incidents	Respiratory incidents	Material incidents	Type of ventilation N° IHT/staff
Doring [10] (1999)	Prospective	35 ICU Neurosurgery	35	Diagnostic	ICHT = NR No serious AEs	Ordinary hypotension = 54% Hypotension <90 mmHg = 2%	Hypoxia n = 10	33%	MV = 65% Doctor n = 1/35

Shirley [18] (2001)	Retrospective	78 ICU	78	Diagnostic	59%	Average BP variation = 17%	NR	Equipment = 37% Organisation = 23%	Junior = 42% Senior = 55%

Lovell [15] (2001)	Prospective	76 ICU/ED	97	TDM = 83% Angiography = 11% ICU + OT = 3%	62% Death n = 1	Clinical problems = 31%		Equipment + environment = 45%	ManV = 97% Junior = 3% Senior = 97%

Beckmann [7] (2004)	Retrospective	176 ICU	191	Therapeutic Diagnostic	100% Serious AE = 31% Death = 2%	Severe hypotension = 3% CA = 3%	Hypoxia = 11%	Equipment = 39% Organisation = 61%	NR

						Clinical problems = 33%			
								
Damm [9] (2004)	Prospective	64 ICU	123	Therapeutic Diagnostic	54%	Hypotension n = 19 Arrhythmia n = 4 CA n = 2	Hypoxia n = 11 Non-adaptation n = 21 Extubation n = 0	MV problem = 21% O2/elec failure:n = 10 O2 disconnection n = 7	MV = 100% Junior n = 117 Senior n = 6

Gillmann [12] (2006)	Prospective Retrospective	290 ED	290	ICU	22.2% Hypothermia = 7% (<35°C n = 20)	6% VF n = 1, CA/AF n = 1 Asystole n = 1	Hypoxia n = 1	Equipment = 9% Uncharged batteries = 4.5% Patient mix-up = 1% Delay = 38%	MV = 65% NR

						Clinical problems = 26%			
								
Lahner [14] (2007)	Prospective	226 ICU	452	Diagnostic = 70% Therapeutic	Serious AE = 4.2%	Asystole n = 2	Bronchospasm n = 1	Equipment = 10.4%	MV = 70% Junior n = NR Senior n >90

						Clinical problems = 26.2%			
								
Papson [16] (2007)	Prospective	297 ED	339	Therapeutic Diagnostic	67.9% Serious AE = 8.9% ICHT n = 4	Hypotension++ n = 6 CA n = 3	OI n = 4 PNO n = 1	Equipment = 45.9% Line = 25.8% Organisation = 2.2%	MV = 72.6% Junior n = 118 Senior n = 221

AE, adverse event; AF, atrial fibrillation; BP, blood pressure; CA, cardiac arrest; ED, emergency department; ICHT, intracranial hypertension; ICU, intensive care unit; ManV, manual ventilation; MV, mechanical ventilation; NR, not reported; OI, orotracheal intubation; OT, operating theatre; PNO, pneumothorax; TDM, tomodensitometry; VF, ventricular fibrillation.

## Discussion

### Physiological impact of transport

Transport impacts on critically ill patients via two main mechanisms. On the one hand, movement of the patient during transport, acceleration and deceleration, changes in posture, and movement from one surface to another are all variables with potential haemodynamic, respiratory, neurological, psychological, and algesic repercussions [5,12,24]. On the other hand, the change in environment from the protection of the initial care unit, equipment changes (ventilator, and so on), noise, the hardness of the examining table and the procedure itself are all sources of extra discomfort [25], and generate additional physiological stress in critically ill patients [24].

These two components must be anticipated and managed at all costs both before and during transport (stabilise the patient beforehand, anticipate sedation) in order to limit the onset of any physiological decline that may lead to an AE (patient-related or otherwise).

### Definitions and types of adverse event

Out of the eight studies, only those by Lahner and Papson [14,16] differentiate between minor AEs (physiological decline of more than 20% compared to clinical status before transport, or problem due to equipment), and serious AEs, which put the patient's life at risk and require urgent therapeutic intervention. According to Papson [16], therapeutic intervention is necessary in around 80% of AEs (minor or serious).

**Figure 1:**
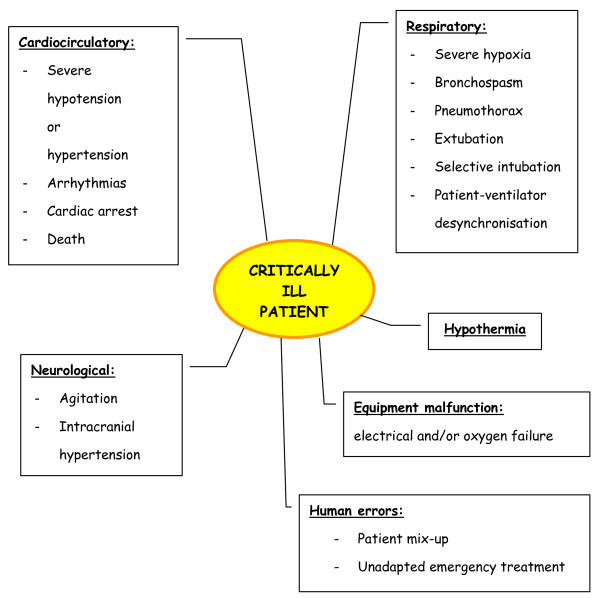
**Main serious adverse events identified since 2004 in studies by Lahner **[14]**, Papson **[16]**, Beckmann **[7]**, Damm **[9]**and Gillman **[12].

**Figure 2:**
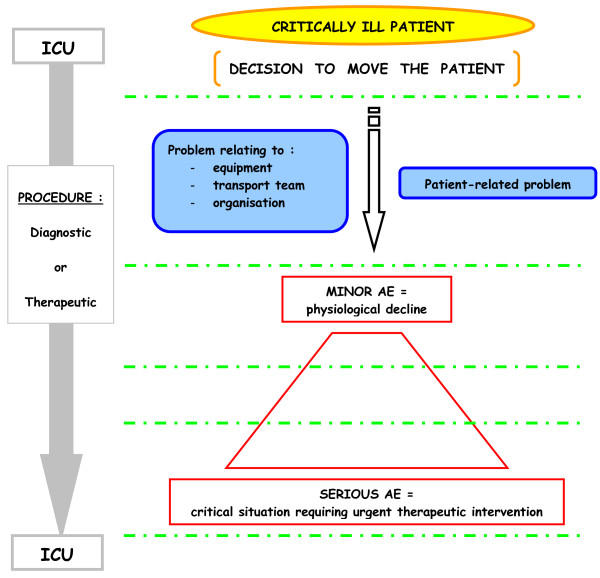
**A comprehensive illustration of the several circumstances leading to a minor and then to serious AE during IHT**. Dashed green lines: Regular checks and corrective action guided by a check-list before, during and after IHT. AE, adverse event; ICU, intensive care unit; IHT, intra-hospital transport.

Figure 1 shows the main AEs that have been identified since 2004 in studies by Lahner [14], Papson [16], Beckmann [7], Damm [9] and Gillman [12]. There also remains a lack of clarity surrounding the causal links between AEs and factors such as patient pathology, equipment, environment and transport management. Figure 2 is a comprehensive illustration of the several circumstances leading to a minor or then to a serious AE, and summarises the *actors *involved in the problem. It is also still difficult to stipulate whether physiological changes are due to transport or the unstable state of the patient [12,19,24,26].

### Adverse event incidence according to the studies

The global incidence of AEs (serious or otherwise) has been known to reach 68% [16], but if only serious AEs requiring therapeutic intervention are taken into account, the incidence ranges from 4.2% to 8.9% [14,16]. In addition, cardiac arrest ranges from 0.34% to 1.6% in the different studies [9,12,14,16].

Beckmann's study [7] identifies serious AEs in 31% of cases including four deaths out of 191 IHTs, but the study only investigates equipment- and organisation-related AEs. This study [7] is a collection of data based on an Australian system of reporting AEs that occur in the anaesthesia-ICU setting (Australian Incidents Monitoring Study: AIMS) [17]. It is based on the voluntary information offered by healthcare givers; a formal evaluation of AE incidence has therefore not been possible since this data collection probably minimize the overall rate of AE.

The global and specific incidence in each study is summarised in Table 1. Risk analysis and the comparison of AE incidence are complicated since there are a number of differences between the various studies [7,9,10,12,14-16,18] with regard to where the patient was admitted, the degree of urgency, the transport equipment, the study population, and the definition of an AE. For example, for equipment-related AEs, certain studies do not acknowledge the nuance between a dislodged oxygen saturation probe and a dropped ventilator [16], or between an untimely ventilator alarm and oxygen failure [9] or even accidental extubation [7]. Given the absence of any clear definitions, it is not possible to standardise results. The specific incidence of AEs associated to a clinical decline ranges from 17% to 33% and is characterised by hypotension, arrhythmia [9,10,18], hypoxia due to ventilator desynchronisation or otherwise [7,9,10], and an increase in intracranial hypertension (ICHT) [16]. The specific incidence of equipment- and organisation-related AEs is between 10.4% and almost 72% according to previous studies [14,16].

Risk factors (RFs) for the onset of AEs, however, are more clearly categorised.

### IHT-related risk factors

Most of the RFs described in the studies do not have any significant statistical value and are usually based on the good clinical sense of the authors [21]. However, according to the studies analysed [7,9,10,12,14-16,18], RFs can be classified into four distinct categories. RFs relating to transport equipment, team and organisation are the most common, whereas those linked to patients and the severity of their clinical status appear to be minimal.

#### Equipment-related risk factors (technical factors)

The three most recent studies involve cohorts of around 300 patients [12,14,16], about 70% of which are on mechanical ventilation (MV).

Damm's study [9] found that around 22% of IHTs involve AEs relating to portable ventilators (one-third untimely alarms and one-third gas or electrical failure). Inadequate know-how and the need for more accurate settings on turbine ventilators might explain the regular occurrence of the associated AEs.

Beckmann's study [7] also highlights the specific risks of MV and upper airway management during transport such as insufficient oxygen reserves, inadequate MV settings, obstruction, malpositioning of artificial airways and accidental extubation. Damm [9] also identified patient agitation and poorly-adapted ventilator settings in 26% of patients, whereas Lovell [15] only found these in 5% of cases. Papson's study [16] demonstrates that equipment problems (in one-fourth of cases relating to tubes, drainage or monitoring lines, and in over half of cases relating to ventilation and artificial airways) are the main cause of minor AEs. Doring [10] identified a link between the number of infusions and infusion pumps, and the onset of equipment-related AEs.

In total, the number of infusion lines [10,16], MV [7,9,14] (change of ventilator or ventilation settings), and sedation [9,10] (initiation, maintenance, modifications) are frequently identified as equipment-related RFs.

#### Risk factors relating to the transport team (human factors)

The IHTs analysed most often involved a team including a junior or senior doctor [7,9,10,12,14-16,18]. Beckmann's study [7] found that certain AEs were caused by a lack of supervision on the part of the transport team, which emphasised their lack of training.

In Papson's study [16] patients were recruited in the ED, and were therefore all transported in the emergency context. The study found that AE incidence is inversely proportional to the doctor's level of experience (junior vs. senior). Lahner [14] on the other hand did not find any increase in AE incidence amongst junior doctors. The explanation put forth by the authors is that the doctors in charge of the IHTs (both junior and senior) had received adequate training, and that the equipment used (such as end tidal CO2 (ETCO2) monitors) had been adapted for transport purposes. These measures allowed them to obtain the lowest AE incidence rates for equipment (10.4%) and serious AEs (4.2%).

#### Risk factors relating to transport indication and organisation (collective factors)

Beckmann's study [7] reports that the majority of equipment- and organisation-related AEs occur during the transfer from ICU to radiology or the operating theatre for diagnostic testing. Communication between ICU and sites of destination or origin is vital for reducing waiting time and therefore transport time [7,15], which was also one of the risk factors identified by Doring [10] for the onset of equipment-related AEs. Damm [9] confirms that AEs are more likely to occur when diagnostic testing (particularly TDM) is required. Hasty transport organisation in the emergency context also leads to the onset of AEs [7]. Gillmann [12] investigated the average waiting time for a patient being transferred from the ED to ICU. Thirty-eight percent of transfers took over 20 minutes to organise, and 14% took over an hour. In almost one-third of cases, the delay was caused by a shortage of available beds. However, according to this study, there is no correlation between waiting time and the onset of complications such as hypothermia. Lahner [14] states that the number of escorts, the destination site (diagnostic or therapeutic procedures), the duration, multiple transfers, and whether the transport took place during the day or night are not factors relating to an increase in AEs. In addition, neither Lahner [14] nor Lovell [15] found any differences in the frequency of equipment-related AEs in pre-arranged transport compared to emergency transport. The differences between the various studies with regard to patient recruitment (surgical, medical, site of origin) and the destination site (imaging, interventional radiology, operating theatre) go some way in explaining why the emergency context is not always identified as a RF.

The duration [7,10] and coordination of IHT [7,9,15], and the associated urgency (haste) [7] therefore vary according to the authors but remain frequently cited as RFs relating to transport organisation.

#### Patient-related risk factors (including clinical instability)

Beckmann's study shows that 42.5% of AEs occur when the IHT is carried out during the initial admission period (in the emergency context when the patient's condition is rapidly changing) or following a recent destabilisation of the patient's condition. Lahner [14] found that there is a link between the severity of the patient's condition (evaluated by acute physiology and chronic health evaluation (APACHE) II score) and minor AEs, but that this is not involved in the onset of serious AEs. Conversely, global AE incidence increased considerably (particularly AEs relating to clinical instability) when transport was carried out in emergency conditions as opposed to being pre-arranged (7.8% versus 2.4% respectively, *P *< 0.05). Papson [16] states that the gravity of the patient's condition is the main cause of serious AEs, but recruitment in his study was exclusively carried out in the emergency context with patients who may or may not have been recently stabilised and were then transferred to theatre or radiology. According to Doring [10] the APACHE III score, therapeutic intervention scoring system (TISS) score, Glasgow Coma scale and the level of urgency are not equipment-related AE risk factors.

The seriousness of the patient's condition is identified as a RF in five out of eight studies. The number of infusion pumps [10], in particular the use of catecholamines [14,15] and positive end expiratory pressure (PEEP) [9,14], and the emergency context (patient instability) [14,16] all lead to an increased risk of AE onset during IHT.

Although many RFs relating to equipment and human management have been identified, there are usually multiple factors involved in the onset of AEs [7]. It is clear that critically ill patients needing to be prepared for transport are at high risk of physiological decline due to equipment (technical factors) and/or clinical status (patient factor), not to mention the collective and human factors that can also intervene [27].

### Secondary effects of IHT

IHTs are suspected of causing ventilator associated pneumonia (VAP) [28], making an active check for VAP necessary in the days following transport. However, patients transported for diagnostic or therapeutic procedures are often more fragile and more at risk of developing VAP anyway. A second study [29] identified age >43 years and fraction of inspired oxygen (FIO2) >0.5 as predictors of respiratory deterioration during IHT.

Morbidity caused by IHT, the length of hospitalisation, neuro-psychological sequellae, and mortality rate are all factors that remain poorly documented. Further clinical studies are necessary in order to evaluate their incidence, nature and severity in the short-, medium- and long-term.

### Preventive measures

Since 1999, in five different countries, IHT has been the object of specific recommendations based essentially on the feedback from experiments and the opinions of experts [4-6,11,21,23]. The various intensive care and emergency medicine colleges and societies have all put forward an almost identical schema for managing patients during IHT in order to improve their comfort and safety. The action plan often presented involves stabilisation of the patient beforehand thus bringing him/her as near as possible to a state of physiological homeostasis, coordination and detailed communication between professionals, and training and experience adapted to the type of IHT (patient with intra-aortic balloon counterpulsation, for example). The equipment must be adapted for transport purposes and facilitate a continuum of care and monitoring during IHT. A form detailing the indication for transport and data on the status of the patient before, during and after IHT is an integral part of the patient's medical file. These recommendations also suggest that an evaluation of transport practices should be regularly undertaken in order to evaluate the quality of critically-ill-patient management during IHT. The European Society of Intensive Care Medicine has issued specific recommendations for the IHT of patients with severe head trauma [11]. British [4,5,21,23] and Italian [6] colleges have also both published specific recommendations for IHT.

Several authors have identified effective *protective *factors for limiting AEs such as regular patient and equipment checks during IHT [7], meticulous preparation of the patient, adapted sedation [7,9], a specialised and experienced escort [7,16], correct use of protocols [7,16,18] and diagnostic and therapeutic units that are located within easy reach of the ED or ICU [7,16].

#### Experience gained from inter-hospital transfers

Over the last 20 years, several authors have investigated the complications involved in IHT [27,30], and have concluded that IHT should be considered as a type of secondary inter-hospital transfer so that management of critically-ill patients is conducted in the same way [31-33]. According to a recent review in the literature on the inter-hospital transport of critically-ill patients, the number of AEs is negligible, and no incidence rate has been established [34]. According to the authors, patients transferred between hospitals are in a less serious condition than patients transferred within hospitals, and they are accompanied by more experienced medical teams, with better transport organisation and management. Several studies [35-37] have shown that, regardless of the severity or degree of organ failure, inter-hospital transfers are safe provided that the accompanying team is experienced and the equipment has been adapted for transport purposes. For both inter- and intra-hospital transport, the level of proof for the identified RFs is low [22,37]. Nevertheless, it has emerged that patient-related RFs rarely intervene in inter-hospital transfers [34]. Better management of factors relating to organisation, equipment and the transport team may therefore be the best way to overcome the risks [34,37].

Inter-hospital transport was the first to revolutionise its practices by recommending that the patient is stabilised beforehand, and that the transfer is carried out by specialist teams [38-42].

#### Efficiency of IHT: Transport indication and risk-benefit analysis

A risk-benefit analysis must be carried out beforehand. In cases involving diagnostic, therapeutic or prognostic modifications, the benefits of transporting critically-ill patients has not been re-evaluated since Caruana's study [8], which identified treatment changes in 24% to 39% of cases in the 48 hours following diagnostic testing.

The development of technology [13] allowing diagnostic (echography, TDM, endoscopy) [43-46] and/or therapeutic (tracheotomy, gastrostomy, laparoscopy, surgery) [47-51] procedures at bedside has contributed to reducing patient exposure to transport-related risks, which is usually unavoidable when carrying out these procedures outside of ICU. The benefits of moving the patient have therefore definitely evolved and merit re-evaluation.

Despite this, certain complementary medical examinations and specialised procedures requiring heavier apparatus (MRI, interventional radiology, theatre) remain indispensable. IHT and its impact on the patient can therefore not be permanently avoided.

#### Stabilisation and preparation of critically-ill patients before IHT

According to most recent studies on IHT, if the patient has been stabilised beforehand, the patient factor rarely intervenes directly in IHT-related AEs [7,14-16,52].

#### Anticipation, organisation and planning of IHT

Anticipation plays a key role in the management of critically-ill patients during IHT [4-6,21,23]. Anticipating a deterioration in a patient's condition (additional preparation before transport), ensuring adequate oxygen reserves and a sufficient number of transport escorts, checking that the retrieval team and the destination site are operational (wall suction unit, oxygen connectors, defibrillator, extension cables, sufficient space for the transport staff to move the patient), and ready to receive the patient in optimal conditions, are also vital prerequisites. The latest studies on patients during IHT show that many complications associated with equipment and collective and human management could have been anticipated [7,15,16,52].

#### Competence of IHT teams

The Australian system of reporting AEs that occur in the anaesthesia-ICU setting (AIMS) [17] reported that 83% of AEs were the result of human error [15].

For patients on MV, risk prevention mainly depends on the competence of the escorting doctor: upper airway management (securing and correct positioning of artificial airway) [4,7,21], adequate ventilator settings (tested prior to departure: FiO2, PEEP, respiratory frequency, exhaled tidal volume (VTE), airway pressure and disconnection alarms) [4-6,11,14,21,23,30,53,54], estimation of a sufficient quantity of oxygen for the entire transport duration with a 30-minute reserve [5,6,11,21,23] (bearing in mind that pneumatic ventilators require at least 50 bars to deliver a tidal volume, and that with turbine ventilators, a 1 m^3 ^cylinder may only be able to independently supply pure oxygen for less than 30 minutes [9]), use of a portable suction unit or an available one at the destination site [4,5,11], monitoring of ETCO2 and interpretation of capnograms [4-6,11,14,21,23] (57% of patients had an ETCO2 monitor during diagnostic testing in Lovell's study [15]), and optimisation of sedation or even curarisation of the patient according to their clinical status [4,11,23] (Damm links patient agitation and poor adaptation to the ventilator with the absence of an inspiratory trigger and a sedation level that has not been adapted for patient transport [9]).

#### Adapted transport equipment

Various pieces of equipment for improving IHT preparation have been evaluated [55,56]. One particular stretcher (life support for trauma and transportation) used for the first time by the military, which integrates the majority of life-support devices and monitoring systems (ventilator, defibrillator, blood gasometry, infusion pumps) has been evaluated for the transport of civilian patients. Although IHT preparation time and the number of escorting personnel are significantly reduced, AE incidence is no different to using the classic type of stretcher.

The US Food and Drug Administration's approval of portable ventilators in 2001 enabled mechanical ventilators to replace manual ones in up to 97% of IHTs in certain establishments [15]. MV during IHTs has shown its superiority over manual ventilation [57] in terms of oxygenation, constant tidal volume delivery, and regular respiratory cycles. However, a bench study analysis of several portable ventilators [58] revealed their inferiority compared to ICU ventilators, particularly due to the differences between their triggering systems, trapped volumes and their difficulty in maintaining a tidal volume. The choice of portable ventilator impacts on the patients chances of adapting and the level of sedation used.

AEs relating to the electrical breakdown (uncharged batteries) of cardio-respiratory monitoring equipment, ventilators or infusion pumps are often found [7,9,16]. Current recommendations advise new generation long lasting batteries (lithium), equipment tracking and maintenance, continuous charging, a sounding alarm in the case of weak battery life, and connecting the transport equipment to wall sockets as soon as possible [4-6,21,23].

A system for securing lines and leads has been proposed in order to limit tangles and knots that often form during patient transport [16].

#### Standardisation of practices - specific protocols for managing IHT

Given the contradictory results and low level of proof in clinical studies on IHT [24], intensive care and emergency medicine colleges and societies have updated their recommendations since 1999, thus providing clinicians with a set of general principles for the good practice of IHT [4-6,11,21,23]. These recommendations represent a first step forwards in the improvement of patient safety and comfort during IHT, and their dissemination seems to have been fruitful since AE incidence during IHT has been on the decrease over the last decade [7,9,10,12,15,18]. However, in studies by Lahner and Papson in 2007 [14,16], the evaluation of serious AEs was unsatisfactory and brings to light the fact that the risks remain real (Table 1). Other prevention measures therefore need to be put into place.

Lahner and Gillman [12,14] conclude that low AE incidence in their studies (≤ 40%) reflects the fact that their escorting doctors had a certain level of education and experience. One of the risk factors identified by Beckmann [7] was inadequate protocols for patient management during IHT, leading to haste and inattention by the transport teams, which probably led to non-observance of recommended IHT procedures. The author thereby emphasises the need for regular equipment and patient checks, and adherence to the protocols that have been put into place to limit AEs. Unlike Doring, Lovell and Damm [9,10,15], Lahner [14] found a link between IHTs carried out in the emergency context and AE onset, which is probably due to the lack of time for optimal stabilisation of the patient, and a lack of equipment checks before transport. The use of a systematic quick check-list for preparing patients for transport might enable teams to remember certain points that may otherwise have been forgotten.

#### Check lists - systematic and final check points

Management protocols which are either too vague or too exhaustive contribute to deviance or straying from practices for managing critically ill patients during IHT [33,52]. Furthermore, accidents are generally preceded by other less serious events that have been ignored (Figure 2). These occur as a result of the association of human, individual or collective errors, with latent or system errors, all relating to the organisation and structure of care units [59]. The next step for reducing the morbimortality of IHT must lead to a method involving strict adherence to issued guidelines [16,22,52].

The field of anaesthesia has already been inspired by current evaluation methods and safety standards in the electro-nuclear industry and civil aviation [60]. More recently, a multicentric, international study involving several university surgical departments [61] evaluated the systematic use of a check-list in the operating theatre (containing nine essential anaesthetic and surgical check points), designed to improve communication within the team and the quality of care delivered to patients. A significant reduction in mortality rate and post-operative complications was demonstrated following the implementation of this check-list.

The use of a check-list which summarises the main points that need to be verified before, during and after IHT may help to reinforce adherence to the recommendations and to further improve IHT management [5,16]. Several authors recommend the implementation of local standardised procedures which are specific to each establishment [4-6,11,15,18,21,23,62], and point out the potential benefits of check-lists [4-7,14,16,18,20,21,24,34,52,62-66] for minimising complications arising from transport, particularly since *re-checking *has been known to limit 91% of AEs [7]. However, our research identified few practical and immediately applicable check-lists for IHT within ICU [4,6,7]. Beckmann [7] puts forward a list of recommendations for helping to prepare patients for IHT, but this is not directly applicable in practice since it contains general precautions rather than relevant detailed check points [52].

Based on our own experience, and having studied a range of international publications on IHT [5,7,9,10,12,14-16,21,23], we propose a list of the main check points and steps that need to be taken before, during and after IHT. This quick, practical check-list (Additional file 1) contains a systematic list of final check points for before and after critically ill patients are moved, and includes: 1) systematic tasks to be carried out before each patient is transported, and 2) systematic patient and equipment checks (ABCDEF) to be carried out after each patient is moved, which focus on the essential points. This check-list only contains pragmatic aspects and avoids being too specific or too vague. It can be carried out quickly at the bedside, especially when the decision to transport the patient has been made in an emergency context. The adoption of this check-list by nursing and medical teams as well as hospital porters and retrieval teams (radiology, theatre) will also be a determining factor in its application and in the quality of the results. Simulation training would be appropriate for implementing and validating competency acquisition for transporting critically ill patients.

## Conclusions

Good clinical sense and a risk-benefit analysis are the only current criteria for deciding on IHT. A sedated, haemodynamically unstable patient on MV, prepared and accompanied by an inexperienced team is a particularly risky combination.

Preparation and management are both crucial steps when transporting critically ill patients since they have a direct impact on the short- and medium-term prognosis of the patient. Having stabilised the critically ill patient before transport, technical, organisational and human factors must be the first targets for the primary prevention of IHT-related AEs. The creation of an IHT-monitoring database would enable the extent of the problem to be measured since, at the moment, not all AEs are declared. A system which tracks monitoring and automatically transfers data to the patient file would enable a real evaluation of the haemodynamic and respiratory changes that occur.

Overcoming the risks of IHT involves taking corrective action for all the causes, and applying methods that have been proven to work in other sectors of activity. A more widespread use of check-lists and proper training plans for teams are also expected to lead to an increase in IHT safety and a lowering of risks in the long-term.

## Key messages

• The IHT of critically-ill patients still involves considerable risk and AE incidence remains high.

• Adapted IHT equipment and comprehensive training programs for all personnel involved are crucial for ensuring that risk factors are correctly anticipated and managed.

• Providing ICUs and EDs with standardised procedures in the form of a check-list constitutes a significant step towards reducing the number of IHT-related AEs.

## Abbreviations

AE: adverse event; ED: emergency department; ETCO2: end tidal carbon dioxide; FiCO2: fraction of inspired oxygen; ICHT: intracranial hypertension; ICU: intensive care unit; IHT: intrahospital transport; MV: mechanical ventilation; PAMV: pneumopathy acquired under mechanical ventilation; PEEP: positive end expiratory pressure; RF: risk factor; TDM: tomodensitometry; VAP: ventilator associated pneumonia; VTE: exhaled tidal volume.

## Competing interests

Dr Gilles Capellier received funding from Resmed company to attend a conference. The other authors declare that they have no competing interests.

## Authors' contributions

All authors conceived the study, and participated in its design. BF and GC performed the literature search and abstracted the data. BF wrote the first draft of the manuscript, which was then revised for intellectually important content by all authors. All authors read and approved the final manuscript.

## Supplementary Material

Additional file 1 **Checklist**. Quick checklist for the intra-hospital transport of critically ill patients. Click here for file
