# Supplementary material for: Recommendations for the intra-hospital transport of critically ill patients
Source: Crit Care. 2010 May 14;14(3):R87. doi: 10.1186/cc9018 (PMC2911721; doi:10.1186/cc9018)
Supplement: Additional file 1 — Checklist. Quick checklist for the intra-hospital transport of critically ill patients. [file cc9018-S1.DOC]

**QUICK CHECKLIST FOR THE INTRA-HOSPITAL TRANSPORT OF CRITICALLY ILL PATIENTS**

**Systematic check points for before each patient is transported:**

EQUIPMENT / PREPARING THE PATIENT:

- Patient labels
- Preparation and equipment adapted to the procedure (MRI)
- Sufficient medication, O2 and electrical reserves
- Breathing:
- Intubation secured and position confirmed on chest X-ray (distance from dental arch = … cm)
- Mechanical ventilation adapted to the patient (alarm and monitoring of tidal volume and insufflation pressure, trigger)
- Intubation equipment, bag + valve + mask, portable aspirator + suction catheters, SpO2, ETCO2
- Circulation:
- Route for venous access isolated and secured (quick injection, administration of vasopressors)
- Medication (emergency, sedation, analgesia, paralysing agents), fluid loading solutions
- Alarms adjusted and activated (ECG, IAP)
- Lines, cables and drainage tubes (Heimlich chest tube valve, abdomen, bladder) unclamped, functional, secure, untangled (i.e. no crossovers) and transportable

TRANSPORT TEAM

- A minimum of three escorts available (including one experienced doctor who is familiar with the patient’s medical history)

TRANSPORT ORGANISATION

- Confirmation of the timetable for the procedure
- Transport route clear, lifts and emergency room available
- Operational equipment (O2 and electrical supplies, ventilator, aspirator) for continuous treatment at sites of procedure

CLINICAL STABILITY OF PATIENT

- Preparation adapted to the clinical status of each patient:
- Breathing (orotracheal intubation, chest drain, synchronisation with MV etc)
- Circulation: optimised haemodynamics (blood volume, vasopressor), haemostasis
- Neurological status: pupils, GCS, intracranial pressure
- Sedation – analgesia – curarisation - hypothermia: prevention and anticipation
- Breaks stabilised, burns and wounds protected
- Head raised if possible (to prevent ICHT and VAP)

**Systematic check points for AFTER each patient is moved:**

- **A**: Airways = integrity of ventilation system

(intubation in place and connected, ventilation tubes, O2 supply)

- **B**: Breath = bilateral auscultation, insufflation pressure, spirometry, SpO2 and ETCO2
- **C**: Circulation = read monitor, check blood pressure and isolate injection route
- **D**: Disconnect = Plug O2 and electrical supplies into wall socket
- **E**: Eyes = monitors are visible to transport team
- **F**: Fulcrum = check points of support

**Anticipate and take swift corrective action for any physiological deterioration in the patient’s condition.**
